# Supplementary material for: MetaRibo-Seq measures translation in microbiomes
Source: Nat Commun. 2020 Jun 29;11:3268. doi: 10.1038/s41467-020-17081-z (PMC7324362; doi:10.1038/s41467-020-17081-z)
Supplement: Supplementary file 10 — Supplementary Data 7 [file 41467_2020_17081_MOESM10_ESM.zip › File2/Confidence_High_Taxonomy/431640_out.krona.html]

Javascript must be enabled to view this page.

members
magnitude
magnitudeUnassigned
count
unassigned
taxon
rank

431640\_out

3

2
3
superkingdom

1239
3
phylum

3
class
186801

order
3
186802

186803
2
family

genus
2
572511

765821

SRS098571\_contig\_number\_contig-100\_25775.66507
species
1

1520805

SRS148253\_contig\_number\_6291
1
species

541000
1
family

1
genus
1263

1262968
species
1

SRS050422\_contig\_number\_19913
